# The Impact of Social Media Disorder, Family Functioning, and Community Social Disorder on Adolescents’ Psychological Distress: The Mediating Role of Intolerance to Uncertainty

**DOI:** 10.3390/children12070861

**Published:** 2025-06-30

**Authors:** Héctor Morillo-Sarto, Javier Torres-Vallejos, Pablo Usán, Juan Ramón Barrada, Joel Juarros-Basterretxea

**Affiliations:** 1 Department of Psychology and Sociology, Faculty of Education, Universidad de Zaragoza, 50009 Zaragoza, Spain; hmorillo@unizar.es (H.M.-S.); pusan@unizar.es (P.U.); barrada@unizar.es (J.R.B.); 2 Escuela de Psicología, Facultad de Ciencias Sociales y Comunicaciones, Universidad Santo Tomas, Santiago 8370003, Chile; jtorresvallejos@santotomas.cl

**Keywords:** psychological distress, adolescent, social media, family relations, community, intolerance of uncertainty

## Abstract

Background/Objectives: Adolescent levels of psychological distress are strongly influenced by community, individual, social, and family factors. Family functioning, social media use, and community disorder have shown high predictive value for psychological distress during this critical stage of development. However, these relationships are not always direct and are often mediated by individual-level variables, such as intolerance of uncertainty. Adolescent psychological well-being is not solely determined by contextual factors; the coping skills developed during this critical stage also play a significant role. Our study aims to analyze how these factors are directly and indirectly related by developing a predictive model of psychological distress in adolescents. Methods: The study included 908 adolescents (46.9% female) aged between 14 and 21 years (M = 16.29, SD = 1.5). Participants completed self-administered questionnaires in a school setting. Structural equation modeling was used to estimate total, direct, and indirect effects. Results: The model showed a good fit to the data. Social media disorder and family functioning showed statistically significant direct and indirect effects on psychological distress. Social media disorder was associated with higher psychological distress, while positive family functioning was protective. Community social disorder was only indirectly linked to higher psychological distress through the increase of intolerance of uncertainty. Conclusions: Intolerance of uncertainty is a critical predictor of adolescent distress, often overlooked despite its significant mediating role. Direct effects of family functioning and social media use also strongly influence distress levels. Impaired family functioning and community disorder interact bidirectionally, creating a cycle that exacerbates distress. Adolescents in these contexts face compounded negative effects from these reinforcing environments.

## 1. Introduction

Adolescence is a critical period of human development, characterized by substantial and intense physical, emotional, and social changes [1]. This transition often entails an increased vulnerability to psychological distress, particularly manifesting as anxiety and depression [2,3] along with a reduced capacity for emotional regulation [4]. Evidence indicates a worrying rise in distress prevalence and levels in adolescents over the last decades [5]. According to the World Health Organization [6], 20% of adolescents suffer from anxiety and/or depression, a figure that has doubled following the COVID-19 pandemic.

However, psychological distress during adolescence is not a uniform phenomenon, and it should be analyzed and understood from an integrative ecological perspective. This approach considers the interrelations between individual, relational, and community-level factors [6]. Individual-level variables, such as social media use disorder, interacting with relational-level family functioning and community-level neighborhood social disorder, shape adolescents’ well-being [7]. Thus, addressing psychological distress from a more integral or ecological perspective makes it possible to better understand it and identify risk and protective factors across different domains.

At the individual level, social media use constitutes one of the more pivotal aspects of day-to-day life in contemporary society. The ubiquity of social media in adolescents’ lives has transformed the landscape of social interaction and emotional development [8]. Although a significant part of the research has focused primarily on time spent on social media to analyze the effect on psychological distress e.g., [9], contemporary research has also emphasized the necessity of considering the type of use more than the time of use [10,11]. This distinction leads to differentiating social media use from problematic social media use or social media use disorder, the latter referring to dependent-like behavior that serves maladaptive emotional functions such as behavioral avoidance among others [11,12].

Social media use disorder is characterized by behavioral manifestations akin to dependence, including preoccupation, withdrawal, tolerance, emotional avoidance, and conflict, among others [13]. For example, adolescents also use social media as an avoidant coping strategy, leading to short-term relief but long-term dependency and distress [12]. Congruently, social media use disorder has been robustly linked to undesirable outcomes such as psychological distress, feelings of loneliness, and a lack of social support [14,15,16,17,18]. Paradoxically, it can create a cycle where unmet emotional patterns (e.g., family dysfunction) lead to further problematic use, amplifying social isolation and increasing psychological distress.

At the relational level, family functioning is a crucial determinant of adolescents’ psychological adjustment. Family of origin is the primary environment for early socialization, emotional learning, and coping ability development. Adequate family functioning is characterized by warmth, caring, and loving, but also by effective use of resources for problem-solving and open communication [19]. These elements are key for the adaptive and healthy development of adolescents [20,21,22]. Conversely, deficient family functioning marked by conflict, emotional unavailability, and neglect negatively impacts adolescents’ well-being as a source of distress. Furthermore, adolescents who are exposed to a poorly functioning family may either avoid seeking familial help even when needed or internalize deceptive patterns learned in this context [23,24,25].

Family and, thus, adolescents and their well-being are also influenced by community-level factors. The social conditions of the neighborhoods where the adolescents live may contribute to their psychological distress. According to models focused on social disadvantage, daily exposure to socially disordered neighborhoods characterized by violence, drug trafficking, or criminality fosters feelings of fear, unpredictability, and lack of control [26,27]. While this factor is more distal, it shapes the sense of insecurity in their direct environment. This is particularly problematic when neighborhood stressors also increase family tensions, affecting family functioning and weakening support systems that may have long-term consequences for adolescents [28,29,30]. Nevertheless, due to its distal nature, the link between community context and individual outcomes is not always straightforward, and the role of mediating mechanisms has been more emphasized than individual or relational factors [31].

Although each of the revised domains influences adolescents’ psychological health, more contemporary research has emphasized the importance of examining them together, as social media problematic use, family functioning, and community social disorder represent the interacting individual, relational, and community-level developmental factors. Similarly, examining potentially mediating mechanisms, such as coping patterns, is crucial. One such mechanism is intolerance of uncertainty, which is conceptualized as a dispositional trait defined by a tendency to experience worry or discomfort in response to ambiguous situations [32,33]. It is considered a vulnerability factor characterized by elements of heightened reactivity and behavioral inhibition in the face of ambiguity related to psychological health outcomes, including psychological distress [34].

Considering that adolescence is usually characterized by a strong component of uncertainty—identity, relationships, and future—that may be reinforced by contextual vulnerabilities [35], how adolescents deal with uncertainty is a determinant. High intolerance of uncertainty in adolescents has been linked to exaggerated fear responses, maladaptive avoidance behaviors, and increased susceptibility to psychological distress [36,37].

Critically, intolerance of uncertainty may mediate the relationship between individual, relational, and community-level vulnerabilities, exacerbating their effect on psychological distress. For instance, adolescents with previous maladaptive use of social media who live in a poorly functioning family and socially disordered community may develop heightened intolerance of uncertainty as a consequence of the chronic unpredictability of their developmental context, reduced perceived control, and increased emotional dysregulation. This heightened intolerance of uncertainty makes adolescents more vulnerable to psychological distress. Despite its relevance, the intolerance of uncertainty on psychological distress remained understudied in adolescents and within integrative ecological frameworks.

### The Current Research

Based on an ecological approach, the aim of the current research was twofold; first, we aimed to analyze the effect of three critical variables: social media problematic use (individual level), family functioning (relational level), and community social disorder (community level) on adolescents’ psychological distress when considered together. Second, we aimed to assess the mediating effect of intolerance of uncertainty on the association between the individual, relational, and community variables on psychological distress.

## 2. Materials and Methods

### 2.1. Sample

The sample comprised 908 secondary students of the Autonomous Community of Aragón (Spain). Participants were aged between 14 and 21 years (*M* = 16.29; *SD* = 1.5), and 46.9% (*n* = 426) were female. A total of 65.3% (*n* = 593) perceived themselves as of medium socioeconomic level, while 10.2% (*n* = 93) and 19.9% (*n* = 181) reported being of medium-high and medium-low socioeconomic level, respectively. Less than 5% perceived themselves as of high (1.5%, *n* = 14) or low (3%, *n* = 27) socioeconomic level. Considering the population of adolescents, the maximum error associated with our estimates at a 95% confidence level is a tight 3%. Less than 1% of the students declined to participate, and thus, the non-response bias was minimal.

### 2.2. Measurements

Depression Anxiety Stress Scales (DASS-21) [38]: Anxiety and depression subscales were used combined to assess psychological distress. Fourteen items (ɷ = 0.93) composed the scale; seven items were originally designed for anxiety (e.g., “I found myself getting agitated”) and seven for depression (e.g., “I felt that life was meaningless”).

Intolerance of Uncertainty Scale—Short Version—Revised (IUS R) [39]: The unidimensional version was used due to the recent findings demonstrating its one-dimensionality [40]. Twelve items (ɷ = 0.83) measuring prospective (e.g., “Unforeseen events upset me greatly”) and inhibitory (e.g., “When it’s time to act, uncertainty paralyses me”) intolerance of uncertainty composed the scale.

The Social Media Use Disorder Scale—Brief Version (SMUD) [13]: Measures social media use disorder and consists of nine items assessing behavioral problems related to social media use during the past year (ɷ = 0.65; e.g., “During the past year, have you often used social media to escape from negative feelings?”).

APGAR Scale [20]: The scale measures satisfaction with family functioning by five items (ɷ = 0.82) referring to adaptation, partnership, growth, affection, and resolve (e.g., “The level of caring and loving relationships within the family”).

Community Social Disorder Scale [41]: A three-question scale (ɷ = 0.75) referring to socially disordered situations in the community (e.g., violence, drug trafficking, and nightlife).

### 2.3. Procedure

Participants were recruited through convenience sampling in collaboration with eight educational institutions that previously agreed to collaborate in the research. Inclusion criteria specified that participants must (1) have a minimum age of 14 years and (2) possess sufficient cognitive and literacy skills to read, understand, and complete the self-administered questionnaires independently. Aligned with current legal requirements and ethical guidelines, eligible students and their parents/guardians received written information about the study, and written informed consent was obtained prior to participation. Data collection occurred during regular school hours in supervised classroom settings.

Less than 1% of eligible students declined to participate, minimizing potential non-response bias. The final sample ensures a maximum estimation error of 3% at a 95% confidence level for population-level inferences.

The current research was first approved by the Ethics Committee of Aragón (CEICA) (C.I. PI23/379) and the Data Protection Unit of the University of Zaragoza (UPD code: 2023-223).

### 2.4. Data Analysis

To assess the proposed theoretical model, structural equation modeling (SEM) was conducted using Mplus 8.7 [42]. Given the categorical nature of several items and scales, the Weighted Least Squares Means and Variance adjusted (WLSMV) estimator was employed. The analysis estimated total, direct, and indirect effects of social media use disorder, family functioning, and community social disorder on psychological distress, with intolerance of uncertainty specified as a mediating variable.

Model fit was evaluated using commonly recommended indices [43]: the Comparative Fit Index (CFI), with values above 0.95 indicating good fit; the Root Mean Square Error of Approximation (RMSEA), with values below 0.06 considered acceptable; and the Standardized Root Mean Square Residual (SRMR), with values below 0.08 reflecting good fit.

## 3. Results

All the variables of the study showed statistically significant correlations (see Table 1). Except for family functioning, variables were positively correlated. Thus, higher levels of psychological distress were related to higher intolerance of uncertainty, more problematic social media use, deficient family functioning, and community social disorder.

The estimated model fitted well to the data (*χ*2 = 2363.880, df = 849, *p* < 0.001; CFI = 0.95; RMSEA [90% C. I.] = 0.04 [0.04, 0.05]; SRMR = 0.06) and explained the 46.6% of the variance of psychological distress and the 23.9% of intolerance of uncertainty. Social media disorder (β = 0.40, 95% CI [0.32, 0.48]), family functioning (β = −0.34, 95% CI [−0.42, −0.27]) and community social disorder (β = 0.08, 95% CI [0.01, 0.15]) showed statistically significant total effects on psychological distress. The total effect of social media disorder was significantly stronger than the family functioning (Δβ = 0.73, 95% CI [0.49, 0.96]) and community social disorder (Δβ = 0.48, 95% CI [0.21, 0.75]); similarly, the total effect of family functioning was stronger than community social disorder effect (Δβ = −0.25, 95% CI [−0.38, −0.11]).

**Figure 1:**
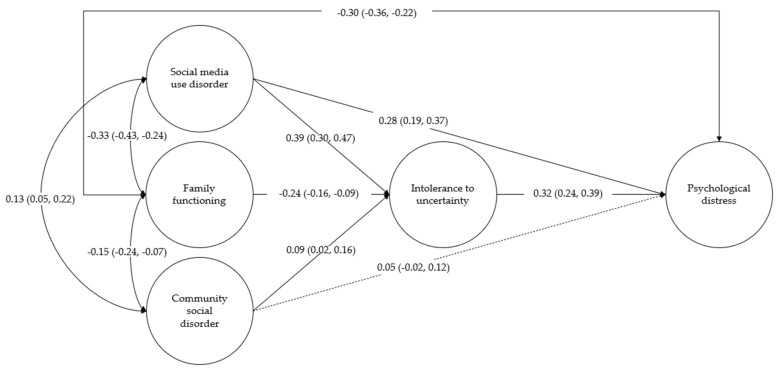
Estimated structural equation model for psychological distress.

When decomposed into direct and indirect effects, different patterns were found for social media disorder, family functioning, and community social disorder. Direct effects are displayed in Figure 1; social media use disorder and family functioning showed statistically significant direct effects on psychological distress, while community social disorder did not. Specifically, higher social media use disorder showed a positive association with psychological distress, and family functioning was negatively associated with psychological distress. The direct effect of social media disorder was significantly stronger than the family functioning effect (Δβ = 0.52, 95% CI [0.34, 0.71]).

Regarding indirect effects, social media disorder (β = 0.19, 95% CI [0.10, 0.28]), family functioning (β = −0.02, 95% CI [−0.03, −0.01]), and community social disorder (β = 0.05, 95% CI [0.01, 0.09]) showed a statistically significant effect on psychological distress through the intolerance of uncertainty. Specifically, social media disorder and community social disorder were positively associated with intolerance of uncertainty, which in turn was positively associated with psychological distress. On the contrary, family functioning was negatively associated with intolerance of uncertainty, reducing ulterior psychological distress (see Figure 1 for direct effects). The indirect effect of social media disorder was significantly stronger than family functioning (Δβ = 0.20, 95% CI [0.11, 0.30]) and community social disorder indirect effects (Δβ = 0.14, 95% CI [0.041, 0.23]). The community social disorder indirect effect on psychological distress was slightly but significantly stronger than the family functioning effect (Δβ = −0.07, 95% CI [−0.11, −0.02]).

## 4. Discussion

Based on an integrative or ecological perspective, this study aimed to analyze the effects of social media disorder (individual level), family functioning (relational level), and community social disorder (community level) on adolescents’ psychological distress, as well as the potential mediating effect of intolerance of uncertainty. Adopting an ecological perspective allowed us to explore how different elements of development interact and shape adolescents’ psychological distress.

Our findings support that different developmental systems of adolescents have a significant impact on their psychological distress but in different ways and magnitudes. The individual-level social media disorder is the more robust predictor of psychological distress, exerting both direct and indirect effects through increasing intolerance of uncertainty. This finding aligns with previous research indicating that problematic social media use is a source of psychological suffering negatively affecting adolescents’ mental health by increasing emotional dysregulation, anxiety, and depression among others [18,44]. As a dependence-like behavior, social media disorder is a maladaptive relationship with social media, where the function of using social media is transformed; in this sense, the use is not for interaction and connection with others but to escape, displacing other activities and becoming a source of preoccupation, conflict, and problems among others [12,13,45,46].

Similarly, our results lie in clarifying other psychological mechanisms underlying the association between social media disorder and psychological distress in adolescence: intolerance of uncertainty. According to previous research, the coping-motivated use of digital media is strongly related to emotional reactions to uncertainty [46] in a potential loop where problematic use fuels intolerance of uncertainty and vice versa. In this regard, the results suggest that social media disorder undermines adolescents’ capability to adaptively face ambiguity, promoting intolerance of uncertainty. One potential explanation is linked to the reinforcement given by social media interactions; due to problematic social media use, adolescents may develop an increased reliance on immediate and predictable feedback (e.g., likes), which is not realistic in offline interactions, undermining their ability to cope with ambiguous and unpredictable contexts. In this case, the lack of compensation in the offline context not only increases psychological distress but also promotes more problematic social media use in an attempt to satisfy these needs [47].

It is also important to note that results indicate that higher levels of social media use disorder were associated with poorer family functioning. This supports the interacting nature of model elements, congruent with previous studies reporting that family dysfunction may drive adolescents to develop maladaptive social media use, while problematic social media use may erode family cohesion [7,48]. Adolescents who did not learn how to adaptively deal with different life events, situations, and difficulties in their family of origin develop unhealthy patterns of interaction, coping, and consumption, like social media disorder, that reduce face-to-face interaction and increase the likelihood of conflictive intra-familial situations.

Beyond its relationship with problematic social media use, family functioning emerged as a key relational factor of adolescents’ psychological maladjustment, both directly and via intolerance of uncertainty. This finding reinforces the broad body of research emphasizing that the family is the primary developmental environment shaping adolescents’ competencies and well-being [25]. The results support that a communicative, supportive, and emotionally available family context buffers adolescents against suffering psychological distress [26,49]. The results obtained here also indicate that adolescents raised in rich family functioning contexts are more likely to learn to adaptively cope with ambiguous situations, which in turn predicts lower levels of psychological distress. These kinds of families tend to be emotionally validating and give immediate comfort, reducing emotional suffering, and they foster internalization and the use of adaptive coping strategies, facilitating the facing of uncertain situations [49].

The protective role of family functioning can be explained in two complementary ways. First, as adolescents’ primary context of development, families with good functioning mechanisms model adolescents’ responses to uncertainty and distressful situations and equip them with competencies to face them. Second, functional families act as secure relational contexts or bases where the adolescents can turn when required (e.g., distress exceeds their coping competencies), reducing uncertainty. This dual protection encompasses internal and external support that can explain the family’s buffering effect [23]. On the contrary, dysfunctional families—inconsistent support, emotional unavailability, etc.—can compromise coping strategy acquisition, while the family context does not offer a secure base to come to when needed. This would make adolescents less tolerant and capable of facing distress and uncertainty, and, at the same time, they would be less likely to seek help in a family context while reinforcing the distress-avoidant response loop [22]. Our results support this, as lower family functioning predicts higher intolerance of uncertainty and psychological distress, but it is also associated with higher social media use disorder.

Our results also suggest that family functioning is not isolated from more distal contextual factors, such as community social disorder, indicating that lower levels of family functioning are associated with higher levels of community disorder. This result is congruent with existing ecological and systemic approaches pointing out the role of family-community interaction relevance on adolescents’ development [30].

This interaction between family functioning and community social disorder may be understood in two non-exclusive ways. As previously shown, adolescents raised in dysfunctional families are more prone to develop maladaptive responses to the context, including heightened emotional distress and intolerance of uncertainty; thus, these adolescents may be more vulnerable to perceiving their neighborhoods as disordered and threatening. Second, socially disordered communities—manifesting crime, drug trafficking, etc.—increase family stress and tension, weakening family dynamics (e.g., reducing emotional availability or fueling conflict) [28,29,30].

Although the community social disorder showed the weakest total effect on psychological distress, it was nonetheless significant; the effect of neighborhood social conditions on psychological distress was fully mediated by intolerance of uncertainty, with its effect slightly higher than the family functioning indirect effect. This result suggests that the community social disorder’s impact on adolescents’ psychological distress operates primarily through their aversive experience when facing uncertain situations. Thus, community social disorder tends to promote maladaptive responses, which in turn increase psychological distress [26,27]. In other words, adolescents living in socially disordered communities are more prone to experiencing psychological distress not for the mere presence of social disorder, but for their perception of lack of control, fear, and derived anxious-inhibitory response to uncertainty [26,27,37].

Our findings also emphasize the importance of subjective interpretation in determining psychological distress. Although adolescents of the same community live in the same material conditions, not all of them respond in the same way to the same objective environment; those with higher intolerance of uncertainty are more vulnerable to experiencing community social disorder as intolerable, triggering higher levels of psychological distress.

### 4.1. Implications for Practice

These findings have important practical implications that lead us to recommend two evidence-informed recommendations. First, our results reinforce the long-standing claim that adolescent development and psychological health must be addressed from an integrative perspective. Interventions targeting only specific-level (e.g., individual) elements may be useful, but they are still insufficient to capture the complex relational nature of factors affecting psychological distress. The observed associations between social media use disorder, family functioning, and community social disorder, and the mediational effect of intolerance of uncertainty, suggest that intervening in any of these levels (e.g., family) may influence others (e.g., social media use).

For example, improving family functioning may buffer adolescents’ vulnerability to psychological distress, reduce problematic social media use, and mitigate the effects of community social disorder. Similarly, public policies to intervene at the community level (e.g., improving neighborhood safety) may reduce the experienced stress by families living in those communities and increase adolescents’ perceived control.

These findings emphasize the interdependence of individual, relational, and community resilience. Integrated interventions simultaneously targeting social media behavior, family functioning, and community social disorder are likely to yield greater benefits for adolescents.

Second, the identification of intolerance of uncertainty as a key mediator mechanism offers clinically relevant insights. Intolerance of uncertainty seems to amplify adolescents’ maladaptive responses to individual, familial, and community stressors, increasing psychological distress. This reinforces its relevance as transdiagnostic vulnerability and underscores the necessity to consider how adolescents face uncertainty explicitly in psychosocial interventions. As a modifiable target, improving adolescents’ competencies for dealing with ambiguous situations may be crucial, especially when triggering individual, relational, and community elements are not accessible to change.

Integrating traditional clinical approaches (e.g., cognitive-behavioral therapy protocols) into school-based interventions where adolescents—and potentially families—are accessible can help improve tolerance to uncertainty of adolescents across different domains.

### 4.2. Strengths and Limitations

This study presents several strengths. It integrates individual, relational, and contextual variables within an ecological framework, offering an integrative perspective on adolescent psychological distress. The use of structural equation modeling allowed for the simultaneous testing of direct and indirect effects, providing a nuanced understanding of the mediating role of intolerance of uncertainty. Additionally, the inclusion of large-scale data from a representative adolescent population within a school context enhances the ecological validity of the findings.

However, several limitations should also be acknowledged. First, due to the cross-sectional design, causality cannot be established. Although the hypothesized pathways are theoretically grounded and consistent with previous research, future longitudinal and experimental studies are necessary to confirm the directionality and temporal sequencing of the observed associations and reinforcing loops. Additionally, the internal consistency of the Social Media Use Disorder Scale in this study was modest (ω = 0.65). While this level is within the range reported in previous validation studies using adolescent samples, it may limit the reliability of the construct’s measurement and should be considered when interpreting the strength of its associations. Second, although diverse in terms of socioeconomic status, the sample was drawn from a specific geographic and cultural context. Future research should aim to replicate these findings in larger and more heterogeneous samples, including adolescents from varied cultural backgrounds and regions. Third, although the study included key variables at each ecological level, other relevant factors were not assessed. For instance, bullying, parental academic support, teachers’ support, and rumination, among others, may play important roles in shaping adolescents’ emotional well-being and should be considered in future models. Lastly, all measures were self-reported, which may introduce biases such as social desirability or shared method variance. The use of multi-informant approaches (e.g., parents, teachers), behavioral data, and longitudinal tracking would strengthen future research.

## 5. Conclusions

In line with the ecological framework, different elements of individual, relational, and community levels are determinants in the explanation of adolescents’ psychological distress. Our results indicate that social media disorder, family functioning, and community social disorder are significant predictors of adolescents’ psychological distress. Similarly, it is pivotal to take into account the mediating role of intolerance of uncertainty. The findings suggest that these developmental systems exert distinct but interconnected effects on adolescent psychological health.

Overall, the results point to multi-factor intervention strategies that address individual, relational, and community risk factors simultaneously. Strengthening family relationships, reducing environmental stressors, and targeting intolerance of uncertainty through evidence-based interventions may offer meaningful pathways to reduce psychological distress and promote adolescent well-being holistically and sustainably.

## Figures and Tables

**Table 1:** Correlation matrix, means, and standard deviations for study variables (N = 908).

Variables	1	2	3	4	5	1
1. Psychological distress	-					
2. Intolerance of uncertainty	0.54 ***	-				
3. Social media use disorder	0.53 ***	0.45 ***	-			
4. Family functioning	−0.49 ***	−0.31 ***	−0.33 ***	-		
5. Community social disorder	0.18 ***	0.17 ***	0.13 **	−0.15 ***	-	
M	9.4	33.2	9.1	12.4	2.2	
SD	8.8	8.3	2.9	2.5	1.9	

** *p* < 0.01; *** *p* < 0.001.

## Data Availability

Research data are not shared. Agreements with participants during the consent process explicitly restrict data sharing to protect their confidentiality and anonymity.
